# Neonatal Male Circumcision: Clearly Beneficial for Public Health or an Ethical Dilemma? A Systematic Review

**DOI:** 10.7759/cureus.54772

**Published:** 2024-02-23

**Authors:** Brian Morris, Beth E Rivin, Mark Sheldon, John N Krieger

**Affiliations:** 1 Faculty of Medicine and Health, The University of Sydney, Sydney, AUS; 2 Schools of Medicine and Public Health, Department of Global Health, University of Washington, Seattle, USA; 3 Bioethics, Uplift International, Seattle, USA; 4 Medical Humanities and Bioethics Program, Feinberg School of Medicine, Chicago, USA; 5 Urology, University of Washington, Seattle, USA

**Keywords:** penile cancer, hygiene genital, lichen sclerosus, balanitis xerotica obliterans, phimosis, bacterial sexually transmitted infections, pediatrics & neonatology, bioethics recommendations, public health policy, male circumcision

## Abstract

Contrasting ethical and legal arguments have been made concerning neonatal male circumcision (NMC) that merit the first systematic review on this topic. We performed PRISMA-compliant keyword searches of PubMed, EMBASE, SCOPUS, LexisNexis, and other databases and identified 61 articles that met the inclusion criteria. In the bibliographies of these articles, we identified 58 more relevant articles and 28 internet items. We found high-quality evidence that NMC is a low-risk procedure that provides immediate and lifetime medical and health benefits and only rarely leads to later adverse effects on sexual function or pleasure. Given this evidence, we conclude that discouraging or denying NMC is unethical from the perspective of the United Nations Convention on the Rights of the Child, which emphasizes the right to health. Further, case law supports the legality of NMC. We found, conversely, that the ethical arguments against NMC rely on distortions of the medical evidence. Thus, NMC, by experienced operators using available safety precautions, appears to be both legal and ethical. Consistent with this conclusion, all of the evidence-based pediatric policies that we reviewed describe NMC as low-risk and beneficial to public health. We calculated that a reduction in NMC in the United States from 80% to 10% would substantially increase the cases of adverse medical conditions. The present findings thus support the evidence-based NMC policy statements and are inconsistent with the non-evidence-based policies that discourage NMC. On balance, the arguments and evidence reviewed here indicate that NMC is a medically beneficial and ethical public health intervention early in life because it reduces suffering, deaths, cases, and costs of treating adverse medical conditions throughout the lifetimes of circumcised individuals.

## Introduction and background

Male circumcision (MC) is a common and long-standing tradition in diverse cultures [1], and the global prevalence is 37-39% [2]. In the United States, the prevalence is 91% among non-Hispanic Whites, 76% among African-Americans, and 44% among Hispanics [3]. By contrast, the prevalence of MC is 10-20% in other Anglophone countries and 10% or less in northern Europe [2]. In Anglophone settings, the procedure is most often performed soon after birth, in which context it is referred to as neonatal male circumcision (NMC).

In recent decades, several health reasons for NMC have been advanced [4]. Thus, NMC may protect against urinary tract infections (UTIs), various sexually transmitted infections (STIs), penile inflammatory conditions (i.e., balanitis, balanoposthitis, and debilitating lichen sclerosis), inferior genital hygiene, phimosis, paraphimosis, sexual problems, penile cancer, prostate cancer, and, in female sexual partners, STIs and cervical cancer (see references in this paragraph). Consequently, NMC contributes considerably to public health. Furthermore, policies and evidence-based reviews by the American Academy of Pediatrics (AAP) in 2012 [5,6] and the US Centers for Disease Control and Prevention (CDC) in 2018 [7,8] concluded that the benefits of NMC outweigh the risks. An independent review of all NMC policies using the AGREE II instrument rated the quality of the AAP’s and CDC’s policies among the highest worldwide [9]. Since then, a systematic review and policy statement by the Circumcision Academy of Australia (CAA) has concluded that the benefits of NMC exceed the risks by a factor of 200 to 1 [10]. All AAP policies expire after five years. Because the strength of the evidence of the benefits and low risks of NMC has increased since 2012, the next AAP NMC policy should provide recommendations aligned with the rising trend in evidence. The increasing strength of the evidence over time was apparent in the difference between the calculation that the benefits exceed the risks by a factor of 100 to 1 in the CDC’s 2018 review [8] and by a factor of 200 to 1 in the CAA’s 2022 review [10].

The AAP policy recommends informing parents and guardians routinely of the benefits and risks associated with NMC before the birth of a child and indicates that the evidence justifies access for the families that choose it, while noting that cultural factors may take precedence for some families [5,6]. The guidelines were deemed helpful by 85.3% of parents/guardians (47.2% white race/ethnicity) in a Miami study [11] and resulted in an increase in NMC from 39% to 58% in a study of 49 (20%) tertiary children’s hospitals in the U.S., in which 49% of those cared for were non-Hispanic white, 24% were African-American, and 27% were “other/unknown” [12].

On the other hand, because NMC involves surgery on the healthy penis of a non-consenting minor, with parents providing proxy consent, and the immediate health benefits to the neonate, though lifelong, are, during the neonatal period, confined to strong protection against urinary tract infections [6,8], it has been argued by some that NMC is unethical, as recognized by the AAP [6] and CDC [8]. Therefore, we performed the first systematic review to address the question of whether, in socio-economically advantaged Anglophone and non-Anglophone European countries, it is ethical for medical practitioners to perform circumcision of boys for non-therapeutic reasons and whether the circumcision of non-consenting minors is legal.

## Review

Materials and methods

Search strategy and study selection criteria. Literature searches were conducted to find articles addressing the issue of whether NMC is beneficial to the immediate and lifelong health and well-being of a male child or whether such an intervention is not only unnecessary but ethical or legal, as such, posing an ethical dilemma for the medical profession and parents of a newborn boy in socioeconomically advantaged countries. The systematic review was conducted in compliance with the 2020 Preferred Reporting Items for Systematic Reviews and Meta-Analyses (PRISMA) guidelines [13], including an update [14] and a related webpage [15]. The methods listed in the latter include inclusion and exclusion criteria, information sources, the risk of bias, and a synthesis of the results. From among the 10 types of systematic reviews [16], we chose the expert opinion/policy review for our systematic review since our focus was on bioethical arguments rather than the synthesis and evaluation of quantitative studies. Such reviews serve the important functions in evidence-based healthcare of complementing empirical evidence or standing alone as the best available evidence. This category of systematic reviews was created in recognition of the need for inclusive and rigorous analyses of reason-based bioethics that have the potential to improve decision-making, both directly and indirectly [17].

Like classical systematic reviews, systematic reviews of bioethical arguments help policymakers make maximally informed and minimally biased clinical and other decisions. A comparator and specific outcome statement are not required for a systematic review of opinion-based literature [16,18]. Systematic reviews of opinion-based literature can, moreover, be incorporated into mixed-methods reviews [19]. Not surprisingly, we found multi-author systematic reviews to be more reliable than single-author narrative reviews. Clinical case studies were particularly prone to bias [20]. The purpose of the present literature review was to present and assess the spectrum of arguments on the ethical and legal aspects of NMC. We did not exclude any article from our sample based on our observation of bias in the argumentation. Several articles have provided further information and advice on the challenges faced when conducting systematic reviews of bioethical arguments [16,21,22], including modification of the standard systematic review process to suit the nature of the bioethics literature (which does not suit the PICO tool format) [20]. According to a previous systematic review, systematic and, especially, semi-systematic reviews of the normative ethics literature on medical topics have become increasingly common [22]. Unlike the present study, many of these reviews do not include a discussion of their limitations.

To identify suitable publications, we searched PubMed, Embase, Scopus, the Cochrane Library, the Philosophers Index, LexisNexis (US and International), and Lexis Nexis (Australia) for articles published in English in the current century, i.e., from January 2, 2000, to August 14, 2023, concerning the ethical and legal aspects of NMC and MC of older children. We excluded from the sample legal cases concerning female genital cutting, sometimes called female circumcision (a misnomer), parental disagreement, non-medical MC (i.e., tribal/traditional MC by unqualified operators), and medical malpractice because these were not relevant to our paradigm, which is whether MC is justified when performed under medical conditions that minimize complications. Our methodology involved sequential searches in that the articles retrieved in earlier searches were not included again in the identification of articles for inclusion in subsequent searches to avoid duplication of articles in the sample.

We first searched PubMed using, in turn, the keywords “circumcision male ethical,” “circumcision male ethics,” and “circumcision male legal.” We next searched Embase using the two-keyword combinations “circumcision” and “medical ethics”, followed by “circumcision” and “legal”, available on the website. We searched Scopus using the keywords “circumcision male ethics” and “circumcision male law.” We searched the Cochrane database using “circumcision”; notably. This database contains only systematic reviews. We searched the Philosopher’s Index using “baby boy circumcision” since this was the most relevant keyword provided by the search tool for that database. Lastly, we searched the legal databases Lexis Nexis (Australia) and LexisNexis (U.S. & International) using the keyword “circumcision.”

We designed the search strategy so that the number of articles included decreased as we progressed through the searches of each database. Next, the titles and abstracts were screened, and then the full text of each potentially suitable article was examined independently by the first and last authors (BJM and JNK) to determine whether the article met the inclusion criteria outlined in the introduction. The reference lists and links to the eligible studies retrieved by the searches were searched to identify additional publications potentially suitable for inclusion. The first and last authors also critically evaluated the articles retrieved to determine whether bias was apparent. Since our focus was on ethical and legal arguments rather than quantitative data and we addressed aims concerning a controversial topic, bias was not only expected but necessary for our discussion of contrasting views, which, as noted, did not affect the inclusion of articles in the sample.

Other methodological considerations. Owing to the considerable heterogeneity in the articles selected and the non-empirical nature of most of them, no standardized quality assessment tool could be applied across the studies. The PRISMA flow chart was made using the most recent template available in the PRISMA 2020 guidelines [13]. The protocol was registered with the International Prospective Register of Systematic Reviews (PROSPERO) and assigned the registration number CRD42022321132. Ethical approval was not required because no animal or human subjects were involved.

Results and discussion

**Figure 1:**
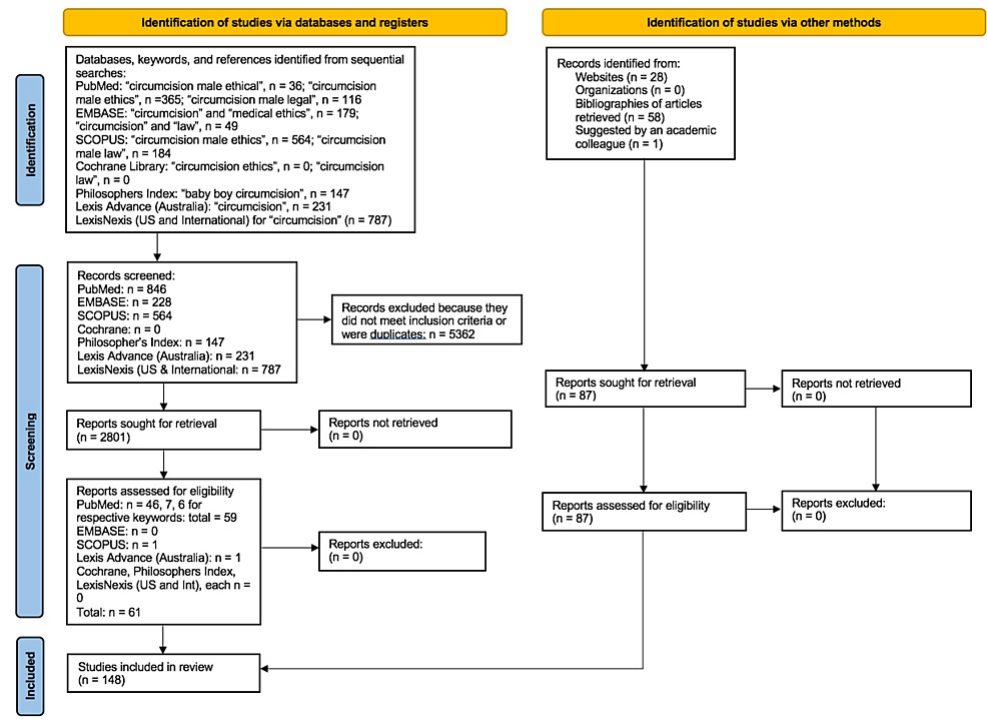
PRISMA flow chart

The search strategy and number retrieved from each database search are summarized in the PRISMA flow chart presented in Figure 1.

Table 1 shows publications retrieved from searches of the databases listed.

**Table 1:** Databases searched and articles retrieved In addition to the 61 references retrieved from database searches, searches of the bibliographies of those articles retrieved an additional 58 references [84-143] and 28 Internet items [70-76]. Another was suggested by an academic law colleague [144]. The grand total of references retrieved from searches was 148.

Databases searched, keywords used for each, total “hits” and number meeting the inclusion criteria				
Database	Keywords	Number of hits	Number included	References
PubMed	circumcision male ethical	355	46	[23-69]
PubMed	circumcision male ethics	354	7	[70-76]
PubMed	circumcision male legal	109	6	[77-82]
EMBASE	circumcision male ethics	186	0	–
EMBASE	circumcision male law	48	0	–
SCOPUS	circumcision male ethics	266	0	–
SCOPUS	circumcision male law	56	0	–
Cochrane	circumcision	0	0	–
Philosopher’s Index	baby boy circumcision	71	0	–
LexisNexis (Australia)	circumcision	262	1	[83]
LexisNexis (US & International)	circumcision	13	0	–
Total number	–	1720	61	61

A cursory examination of these references indicated that a mixed-methods approach [19], owing to its flexibility, was best suited to the aims of the study.

Ethics and human rights

According to some philosophical arguments, the MC of non-consenting minors is a violation of their human rights and bodily integrity [41-43,50,59,134,145]. Further, (1) NMC is ethically and/or legally problematic if the practice has no health benefits or causes sexual problems, and (2) NMC is ethically, morally, and/or legally problematic irrespective of whether the practice has health or sexual benefits. While item (1) can be analyzed and critiqued internally to determine whether arguments are logically valid and sound, for item (2), the issue of health or sexual benefits is irrelevant. The arguments against NMC have sometimes been emotive and lacking in supportive evidence [47,59,85,145,146], as we shall discuss, whereas scholarly assessments by others have reached the opposite conclusions [23,27-29,51,53,70]. Table 2 summarizes the advantages of NMC compared with delaying the procedure to an age when the older boy or man can decide for himself, and Table 3 summarizes the advantages of leaving a boy uncircumcised.﻿ 

**Table 2:** The advantages of NMC as opposed to MC for older boys and men

Neonatal male circumcision	Delay until age of consent
• Is a simple surgical procedure for a competent medical practitioner	• Is a more complex procedure
• Quick: takes several minutes	• Takes 30 minutes or more
• Cost is relatively low	• Much more expensive and can be unaffordable
• Low risk of adverse events (0.4%), most minor	• Higher risk of adverse events (incidence 4–8%)
• Any bleeding is minimal and easily stopped	• Prevalence of bleeding is greater, requiring cautery or other interventions
• Sutures not needed	• Sutures or tissue glue required
• Convenient since the baby sleeps most of the day	• Inconvenient owing to need for time off school or work
• Local anesthesia used if < 2 months old	• General anesthesia for age > 2 months to 9 years. For older ages local anesthesia may be used, although general anesthesia may be preferred by the surgeon
• Healing is fast (< 2 weeks)	• Healing time is ≥ 6 weeks
• Cosmetic outcome usually good	• If stitches are used, stitch marks may be visible permanently
• No adverse psychological effects	• Potential for adverse psychological effects
• Immediate benefit against UTI risk	• Benefits delayed until after the procedure
• Fulfils right to optimum lifetime health	• Postponing until age of consent permits “self-determination”
• Lifetime benefit to risk ratio ≥ 100:1	• Procedural complications may exceed benefits

**Table 3:** Advantages of leaving the male uncircumcised

Benefit to the male of not undergoing circumcision as a child
• Self-determination/autonomy – allows the boy to decide for himself
• Psychological benefit from knowing he made the decision himself
• Avoids risks, however low, from undergoing NMC surgery
• Parental choice to ensure his penis resembled his peers in majority uncircumcised cultures
• May be easier to elicit ejaculation during masturbation or fellatio
• Foreskin available if required for later for skin graft, sex reassignment surgery, or lifestyle sex practices amongst specific sexual minorities.

In 2007, the prominent British journal BMJ published two brief “head-to-head” articles debating NMC. In the first, Dr. Geoff Hinchley, an emergency medicine physician, argued against infant MC in an emotive, one-sided manner based on fallacies [47]. The latter included referring to MC as “male genital mutilation” twice, a discredited study claiming that MC causes “reduced penile sensation in adulthood for all," that NMC is performed “often without anesthetic," and that it provides “no medical benefit." Dr. Kirsten Patrick then argued in favor of the practice based on the “recent strong evidence” that it is “medically beneficial” and “if competently performed, NMC carries little risk," with the pain “if done under local anesthesia [being] comparable to that from an injection for immunization," which also carries risks [60].

Indeed, it has been argued that, given the wide-ranging protection that MC provides against multiple medical conditions and infections in infancy and childhood and STIs in sexually active adolescent males, leaving boys uncircumcised would be unethical [23,51]. Specifically, Article 24 of the United Nations Convention on the Rights of the Child (CRC) states that “States Parties recognize the right of the child to the enjoyment of the highest attainable standard of health… States Parties shall strive to ensure that no child is deprived of his or her right of access to such health care services” [147], which include preventative health care. To the extent that failing to advise parents about the benefits and risks of NMC may be prejudicial to their children’s health and in violation of their rights, we and others have interpreted Article 24 as mandating NMC [51]. 

Lawyer Allan Jacobs, M.D., J.D., when he was an obstetrician/gynecologist at Stonybrook University, and Kavita Arora, M.D., when an obstetrician/gynecologist at Case Western University, offered a three-part test based on the positive and negative impacts of NMC on society and individuals [53]. They concluded that NMC is permissible and that, irrespective of local norms, it does not violate human rights. Robert Darby, Ph.D., an independent researcher and historian, was “happy to agree” with this conclusion, provided that the procedure is “performed hygienically in a hospital by an accredited mohel or other qualified operator, using full anesthesia” [34]. Jacobs and Arora, however, stated that “adequate anesthesia does not necessitate general anesthesia,” which carries inherent risk, and that “the earlier circumcision is performed, the safer it is … [and] … as physicians, we routinely counsel parents regarding the risk versus benefit calculus in making medical and surgical decisions … So, it is of utmost importance to acknowledge the quality of the data in this conversation - a point that is often overlooked by critics of circumcision … [and, for greater safety] … circumcisions [should be] performed during infancy] … [rather than later, when the] … pain, safety, and extent of the procedure” are significant [52].

In an article opposing NMC, Stephen Svoboda, executive director of Attorneys for the Rights of the Child, Peter Adler, Adjunct Professor of International Law at the University of Massachusetts, and Robert Van Howe, a pediatrician in private practice and honorary clinical professor at Michigan State University [64,111], cited a document issued by the International NGO (non-government organization) Council on Violence Against Children (ICVAC) in 2016 [148] in support of their position, though this document devotes only 1 of its 48 pages (page 22) to MC, on which it is observed that the World Health Organization (WHO) supports the procedure for protection against HIV. However, the document makes no mention of the additional wide-ranging medical benefits starting in infancy, instead citing the 2010 Royal Dutch Medical Association’s MC policy, which was developed by medical ethicist Gert van Dijk, M.D., urging the delay of MC until after the age of consent [146], likewise without presenting the scientific evidence supporting NMC. The 2016 ICVAC document also cites reports from Kenya, Norway, and Germany in 2012 and earlier that failed to translate into legislation (as discussed further below).

Opponents of childhood MC have also cited Article 24(3) of the CRC, which recommends that “States Parties shall take all effective and appropriate measures with a view to abolishing traditional practices prejudicial to the health of children” [147]. However, the WHO’s Manual for Early Infant Male Circumcision under Local Anaesthesia [149] supports NMC, as does UNESCO, thus indicating that medically performed MC of minors is consistent with respect for and protection of a child’s right to health. Likewise, academic lawyers Vawda and Maqutu in Durban, R.S.A., argued that “In the face of the compelling evidence available, it would be foolhardy to forego this important intervention [i.e., NMC] in the fight against the rampant HIV/AIDS pandemic. Limiting the rights of neonates under such circumstances can be regarded as a justifiable measure to protect public health.”

Vawda and Maqutu argue that NMC is justifiable as a public health necessity [91]. Stating, “In the face of the compelling evidence available, it would be foolhardy to forego this important intervention in the fight against the rampant HIV/AIDS pandemic. Limiting the rights of neonates under such circumstances can be regarded as a justifiable measure to protect public health.”

The claim that childhood MC is harmful and that children have a “right to health” [114] thus seems contradictory given the favorable risk-benefit ratio of NMC and the high proportion of uncircumcised males affected by adverse medical conditions associated with the foreskin [112,128,150,151]. Public health ethics is primarily consequentialist, informing decisions that are likely to produce the greatest net benefit, and well-informed public health authorities should be persuaded by the strong evidence in favor of NMC to follow the policies of the AAP and CDC in this regard. So also A.M. Viens, Ph.D., when in the Department of Philosophy at Oxford, who argued in 2004 that the case against allowing parents to choose whether NMC is in their children’s best interest has been inconclusive [31].

Autonomy and self-determination

A core argument against NMC concerns a child’s human right to bodily integrity. Jacobs [130] summarized this argument as “To the extent that notions of autonomy are comprehensible, autonomy is held to be a trump card, so that a minor breach of genital physical integrity outweighs all other considerations of right and interest.” A right is a valid claim, while an interest is anything that promotes benefit or avoids harm. Opponents of NMC have argued that NMC does harm, disputing scientific findings and evidence-based policies supporting the rights of parents to choose NMC and describing any benefits as insufficient or realizable in males old enough to decide whether they wish to be circumcised [25,39,43,44,56,59,62,65,72,73,78,82,104,105,115,152]. A systematic review found such claims to be based on speculation and misinformation [76]. For example, claims that MC is a high-risk procedure were contradicted by the low risk of adverse events; claims that NMC causes psychological harm were contradicted by studies showing no such harm; claims that NMC impairs sexual function and pleasure were contradicted by high-quality studies that found no adverse effect; and claims disputing the medical and health benefits of NMC were contradicted by a large body of high-quality evidence indicating at least partial protection against a wide array of adverse medical conditions, infections, and diseases in males of all ages and their female sexual partners when sexually mature. In the current “post-truth” era, vocal minority groups may consider their opinions more important than those of medical and scientific experts [153]. Such attitudes fit with a pattern of radical individualism, devaluation of scientific evidence, and promotion of autonomy.

It can be argued that the rights of the neonate should be balanced. For example, self-determination should be balanced against the right to health. Thus, the child’s best interest should be given more weight than the notion of the neonate’s self-determination. The right to health would include vaccine requirements for school entry and the general response to the COVID-19 pandemic that exemplifies societies’ decision that the right to personal and public health takes precedence over individual rights to self-determination.

According to a statement by the Brussels Collaboration on Genital Integrity [123], an intervention to alter a bodily state is medically necessary when the bodily state poses an immediate serious threat to an individual’s well-being. While not being circumcised may not immediately threaten the health of a child, the evidence indicates that uncircumcised individuals may face unique health threats later in life. Given the degree and breadth of benefits conferred by NMC, we suggest that the procedure is an example of the type of alteration of a bodily state described in the Brussels Collaboration statement. Likewise, under international human rights law as articulated in Article 5 of the CRC [147], “States Parties shall respect the responsibilities, rights, and duties of parents or, where applicable, the members of the extended family or community as provided for by local custom, legal guardians, or other persons legally responsible for the child, to provide, in a manner consistent with the evolving capacities of the child, appropriate direction and guidance in the exercise by the child of the rights recognized in the present Convention.” That is, the extended family or community should decide how to care for children with their best interests in mind.

The cultural context is also obviously important, as the Australian Family Law Council [83] recognized regarding MC in its discussion of “female circumcision,” stating (item 2.54), “In Australia, male circumcision is not unlawful. It has religious significance for persons of particular religious persuasions, such as those of Jewish faith. It is also understood to be performed as an initiation rite for males entering adulthood in some Aboriginal communities.” Members of other cultures and of religions, such as Islam and Coptic Christianity practice MC as a form of group identity. Though some may construe this as arguing from the perspective of tradition, we would argue that NMC should be justified if the procedure is supported by scientific evidence.

Balancing rights can be challenging. However, the right to the highest attainable standard of health, including access to preventive health care, should outweigh other rights [113]. Arguments against NMC based on children’s right to bodily integrity and future autonomy have been refuted by authorities in ethics, who have pointed out flaws in those arguments and argued that NMC falls within the prerogative of parents as a prophylactic measure with unequivocal health benefits that must be performed by competent practitioners using anesthesia [27-29,51,53,70,71,74]. Along these same lines, the present co-author, medical ethicist Mark Sheldon, Ph.D., at Northwestern University, Chicago, argued that “The world in which the issue of harm is raised is ultimately the same world for all, even if social traditions are different” [154]. Medical education academics Brusa and Barilan at Tel Aviv University argued that being circumcised increases rather than constrains autonomy [33]. Along other lines, bioethicist Wim Dekkers argued that “‘bodily integrity’ is an ambiguous notion that cannot be ‘applied’ in practice” and “can be overridden by competing moral obligations, for example, to obey God’s law [in Judaism and Islam] or to contribute to the health of the patient” [37,38]. In evaluating NMC and autonomy, independent researcher Akim McMath argued that “bodily integrity is a prima facie principle in its own right, closely connected with, but fundamentally different from, the principle of personal autonomy” and that “we should be idealists when evaluating the child’s own interests, but realists when evaluating public health justifications for circumcision” [55]. Note that a prima facie principle is one that is valid but that can be overturned by a competing claim of greater strength. The claim can be either intellectually or empirically more powerful.

Legal arguments

The legality of NMC has been extensively evaluated by professors of law, bioethics, medicine, medical sciences, and public health, who have found that the case law provides examples in favor of NMC, that NMC is low-risk, that lifelong benefits accrue immediately, and that consent is required by parents [23,75,102,113]. A review of the legal database Nexis Uni for U.S. state and federal cases identified 77 relating to MC from 1939 through 2021 [69], a minuscule number given the millions of NMCs and medical MCs over this period. The most common reason for the lawsuits was surgical negligence (49%), and most involved patients who were minors. The complications mentioned included aesthetic dissatisfaction (20%), pain (19%), impaired sexual function (17%), and surgical trauma or injury (16%). The verdicts generally favored the physicians (59%). Proving malpractice requires proving causality, i.e., that the breach of satisfactory care caused the injury. This would be difficult. Legal cases, including malpractice litigation and criminal cases, represent isolated reports in which problems occurred. Furthermore, these reports are not scientific but represent a court's evaluation of medical evidence.

Among the legal arguments, Jeffrey Brown, M.D., a member of the AAP Medical Liability and Risk Management Committee [32], argued that physicians may be accountable for not telling parents about the benefits and risks of interventions for their children, including circumcision and vaccination. He pointed to the risk of UTIs in uncircumcised infants and STIs in sexually active minors, as well as the lower risks and costs and higher benefits associated with NMC compared with MC, as further evidence against waiting to make the “circumcision decision.” By contrast, Darby argued for preserving the foreskin at all costs [73]. Sir William Patrick Dean, a High Court judge (and former Governor General of Australia), issued a non-binding opinion that MC “for perceived hygienic - or even religious - reasons … plainly lies within the authority of parents of an incapable child to authorize surgery on the basis of medical advice” [155]. At the time, the medical evidence favoring NMC was not as strong as it is now. Opponents of NMC cited a case of surgical non-therapeutic sterilization (see review [77]) in a warning to doctors that they could face criminal assault charges for performing the procedure [92]. Likewise, when Van Howe provided an affidavit supporting the plaintiff in a lawsuit in Minnesota District Court against Mercy Hospital regarding the medical complications of an NMC [156], the judge characterized his evidence as “confusing … inconsistent and self-contradictory” in dismissing all counts.

Lobbying efforts by opponents of NMC to ban MC of minors in the United States [157] and northern European countries [158,159] have led to congressional and parliamentary debates and legislation upholding the right of families to have their children circumcised safely [121]. To date, the efforts to ban the MC of minors have failed [57,61,160,161]. In 2012, a court in Cologne considered a bleeding complication involving a Muslim doctor who circumcised a Muslim boy [162]. The outcome was misconstrued by international news media, who interpreted it as a ban on MC in Germany [163]. In fact, the court described the legality of circumcision of minors as one of the “undecided questions of law” and found that the defendant was not guilty, with his costs to be paid by the public (see the full English translation [164]). An appeal by the prosecution failed. Notably, in response, the German Ethics Council gave its support to the circumcision of male minors with certain provisos [61]. Taking this advice, the Bundestag (national German parliament) then enacted legislation upholding the legal right of parents to choose circumcision performed by a trained professional in a safe environment for religious or other reasons [161]. Critics claimed the law was political (e.g., [25]). But all legislation is, of course, political. Parentally approved circumcision in a hospital by “knowledgeable staff” is legal in France [79] and virtually all countries.

An academic law organization at the University of Tasmania recommended that non-therapeutic circumcision of boys be banned [165]. An article in BMC Pediatrics by a lawyer and non-Tasmanian academics found the recommendations to be illogical, dangerous, and unworkable and that “doctors should be allowed to perform medical procedures based on sound evidence of effectiveness and safety with guaranteed protection. Parents should be free to act in the best interests of the health of their infant son by having him circumcised should they choose” [23].

As alluded to earlier, MC is sometimes compared to female circumcision, often termed “ritual female genital mutilation” (FGM) [35,37,43,49], with most forms being quite anatomically dissimilar to MC. At least one critic has referred to MC as “male genital mutilation” [47], an emotive term that appears self-serving. Opponents misconstrued [44] an FGM case in the United Kingdom by failing to note that two items (72 and 73) in the judgment by Sir James Munby, President of the Family Division of the High Court of England and Wales, recognized substantial health benefits of childhood MC, unlike FGM [166] (see [80] for a critical evaluation of the judgment).

An Australian family physician, Dr. Terry Russell, argued [167] that “any person who is advised against or denied circumcision on spurious grounds, who then goes on to suffer from one of the conditions which might reasonably have been prevented or minimized by circumcision, has a right to damages against the person who advised against or denied circumcision on spurious grounds." Ethicist Mirko Garasic, when at Monash University, Australia, argued that the permissibility of circumcision of boys “should only be evaluated within its medico-ethical dimension without the political charge often associated with the issue” [45]. It should be noted that the law is national or local, subject to judicial interpretation, and subject to change.

Professional ethics and the Hippocratic Oath

The maxim “First, do no harm” has been widely attributed to Hippocrates but is not found in the Hippocratic Oath. The oath is sometimes referred to by individuals who object to the NMC. It may also influence medical practitioners’ and parents’ views on NMC. Kamran Abbasi, Fellow of the Royal College of Physicians and Editor in Chief of the BMJ, regarded this “ancient oath” as “true in spirit but impossible to practice in the messy business of modern healthcare” [120]. In reality, the maxim is a mistranslation. The English phrase is a direct translation of the Latin phrase primum non nocere used to translate the Greek phrase “ὠφελέειν ή μὴ βλάπτειν,” which actually translates literally as “do benefit or [at least] don’t do harm.” The Hippocratic Treatise on Epidemics (1.5) states, “I will apply the regimens of treatment according to my ability and judgment for the benefit of my patients and protect them from harm and injustice” [125] and “I will prevent disease whenever I can, for prevention is preferable to cure” [89, 168]. NMC thus accords with the Hippocratic Oath.

The followers of Hippocrates were strictly physicians and did not perform surgery. These days, even general practitioners perform minor surgeries such as NMC. Initially, every surgical intervention begins by doing harm, in this case, in the cutting, manipulation, excision, and, for older males, stitching necessary for the procedure. After healing, it is expected that the intervention will prove useful. Rarely is any “harm” from NMC more than minor or temporary. Cases involving harm merit publication because of their rarity. In contrast, approximately half of uncircumcised males suffer at least one foreskin related medical problem during their lifetime [112,128,150,151].

Consent in the form of substituted judgment

Parents and legal guardians should, of course, consent to all beneficial medical therapies and interventions for their children. Substituted judgment by individuals who are fully informed and acting in the demonstrated best interest of individuals who cannot decide for themselves is central to ethical decision-making. Thus, parents are legally permitted to consent to NMC when they are fully informed about the risks and benefits. It should be noted that substituted judgment means doing what the person would have wanted when that person, who was previously competent to decide, no longer has that ability. The substituted judgment standard is therefore not applicable to children, who never expressed valid opinions for parents about what they might want. Distinguishing between rights and interests can be challenging as well. Indeed, it can be challenging to determine whether an assertion of a right or an interest should be generally accepted. Those on opposite sides of the NMC argument conclude differently. The advantage of calling something a right is that it at least partially cuts off discussion. As argued by Mazor [74] and Jacobs [53], a right is a valid claim, while an interest is anything that promotes benefit or avoids harm. Parents must choose which argument to side with in making the “circumcision decision”.

Douglas Diekema, M.D., bioethicist member of the AAP Task Force on Circumcision and author of correspondence for the AAP’s repudiation of an accusation by mostly northern Europeans of cultural bias in the AAP’s policy [98], summarized the issues and consent process [40] used in the AAP’s 2012 policy recommendations [5,6]. The information that medical practitioners must provide to parents before obtaining informed consent for NMC includes (1) a list of common conditions prevented or substantially reduced by NMC that pose a risk to the health and well-being of the boy during infancy and childhood and (2) procedural risks that are low (0.4%) for NMC but are 20 times higher when circumcision is performed between 1 and 10 years and 10 times higher after the age of 10 [100]. Moreover, (3) NMC obviates the need to seek specialist treatment for later foreskin disease; (4) the costs of later MC, often for medical need and for treatment of a wide array of conditions that NMC protects against, are substantially higher than the cost of NMC [54,93,169-173]; (5) the health parity rights of the poor in protecting their children’s health [87,88], while contradicting arguments to the contrary [141], which have been disputed [142]. In the US, states that lack Medicaid coverage have NMC rates 24% points lower than states with Medicare coverage [87].

The issue of consent is, as has been seen, a common argument used by opponents of NMC [59,82]. Alex Myers, B.A. (philosophy), and bioethicist Brian Earp, Ph.D., argued that “medically unnecessary penile circumcision ... should not be performed on individuals who are too young to provide meaningful consent to the procedure” [59] but did not cite systematic reviews of the medical evidence showing the immediate and lifelong benefits and low procedural risks (e.g., [112,128,150,151]. The NMC consent process is based on the principles of mutual trust, shared responsibility, and understanding between physicians and parents, who are provided with a document outlining the procedure, goals, risks, benefits, and expected outcomes [97].

Best time for circumcision

As this discussion makes clear, despite the advantages of NMC over later MC summarized in Table 2, MC at any age requires a well-trained, competent surgeon (who can often be a family physician or general practitioner) and the use of pain relief, preferably local anesthesia. The AAP policy also offered other recommendations in 2012 [5,6].

Consequences of NMC for disease prevalence in Anglophone countries

Here, we estimate that, in the United States, the prevalence of NMC would decrease from an estimated 80% currently [3] to 10%, as in most of Europe, the cases of various adverse lifetime medical conditions per annual U.S. birth cohort of 1.57 million males would increase by more than 1 million (Table 4). This would include multiple cases for some individuals and none for others. In Australia, conversely, if there was an increase in the prevalence of NMC from the level of 19% in 2019 [132] to 90%, it has been calculated that this would result in approximately 77,000 fewer cases of adverse medical conditions over the lifetime of the 2019 annual male birth cohort of 157,000 [131].

**Table 4:** Projected effect of a decrease in the prevalence of NMC in the United States from 80% to the level in much of Europe, 10%, on conditions against which NMC protects *Based on the latest US CDC Health Statistics data of 1,967,458 male births in 2019. #Based on data for circumcised vs. uncircumcised males [112,128,150,151]. ¥The percentage of males who will be affected over their lifetime as a result of the single risk factor of retention of the foreskin. Data for STIs were estimated after taking into account the external factor of heterosexual exposure and the population prevalence of each STI in the U.S., Canada, Australia, the U.K., or other countries. The estimate of the risk reduction conferred by MC was based mostly on U.S. data because more data from the U.S. were available. As an example of how the results were calculated, for prostate cancer, the lifetime risk in the U.S. is 1 in 8 (0.125), so for a 17% risk reduction conferred by early circumcision, according to a major U.S. study [194], the percent affected would be 0.125 x 0.17 x 100 = 2.1%. The more prevalent a condition, the greater the number of males who will be affected over their lifetime. ∂Number of additional cases if NMC prevalence decreased from 80% to 10% was obtained by multiplying the fraction affected (from column 3) by 1,967,458 x (80 – 10)/100 = 1,101,762. HPV: human papillomavirus; HIV: human immunodeficiency virus; K: thousand; M: million; Meta: meta-analysis; OS: original study; RCT: randomized controlled trial; SR: systematic review.

Medical conditions, risk reduction, and number of cases prevented				
Condition	Decrease in risk^#^	Approx. % affected^¥^	Study type [Reference]	Approx. number of cases prevented^∂^
Urinary tract infections: lifetime	72%	27	Meta [174]	420,000
Phimosis persistence at age ≥18 yr	97%	3	SR [175]	47,000
Balanitis	68%	10	Meta [94]	160,000
Candidiasis (thrush)	60%	10	OS [176]	160,000
High-risk HPV infection	60%	10	Meta[177]	160,000
HIV (acquired heterosexually)	72%	0.1	Meta [178]	1,000
Genital ulcer disease	50%	1	OS [179-181]	16,000
Syphilis	50%	1	Meta [182],OS [183]	16,000
Trichomonas vaginalis	50%	1	RCT [184]	16,000
Mycoplasma genitalium	40%	0.5	RCT [185]	8,000
Herpes simplex virus type 2	30%	4	RCTs [186-189]	6,000
Chancroid	50%	1	Meta [182]	1,000
Penile cancer (lifetime)	95%	0.1	OS [190-192]	2,000
Prostate cancer: population-based	17%	2.1	OS [193]	42,000
Totals	–	70	–	1,100,000

Procedural risks

Modern medicine is about achieving the best possible benefit-to-harm ratio. It is universally accepted that the most effective treatments carry the risk of complications. Opponents of NMC emphasize procedural risks, but devastating penile injuries are extremely rare, and mortality is rarer [100,195]. Among newborn males, the study by CDC researchers found amputation of penis was 4 per million among uncircumcised newborns and zero among circumcised [100]. The AAP policy found the minor adverse event frequency to be 0.5%, with almost all injuries being easily treatable with complete resolution [6]. Serious complications requiring hospital admission affected only 0.02%. Similarly, the 2014 study by CDC researchers of 41 possible adverse events in a large administrative claims data set for the 1.4 million procedures performed in the United States found the adverse event frequency was 0.4% for NMC [100], similar to a large 1989 study [195]. The frequencies of serious complications were 20 times higher when the procedure was performed on children between the ages of 1 and 10 and 10 times higher when performed on patients older than 10 years [100]. The CDC cited a 2014 Mayo Clinic Proceedings analysis [150] stating that the benefits of NMC exceed the risks by “100:1” [8].

Sexual pleasure and function

It has been argued that the pain experienced during NMC results in permanent damage to vulnerable neuronal sensory structures in neonates, and, as a result, the sexual experience of neonatally circumcised men is diminished [59]. A recent extensive systematic review of all of the evidence relevant to this argument, including physiological measurements, the location of the sensory receptors, and meta-analyses of sexual dysfunction, concluded that the consensus in the highest-quality literature was that non-therapeutic MC, including NMC and adult circumcision, has minimal or no adverse effects on sexual functions, sensation, satisfaction, and pleasure [196]. However, several functions were found to be significantly better among circumcised men [197], including in RCTs of adult MC [198,199]. The most erogenous regions of the circumcised penis-the glans and distal ventral shaft-have direct contact with the vaginal wall during sexual intercourse, and such contact has been suggested to enhance sexual pleasure [200]. The glans and distal ventral shaft of the uncircumcised penis have direct contact with the vaginal wall during the in-stroke, but during the out-stroke, the retracted skin is compressed into tight folds behind the highly erogenous corona glandis, thereby reducing sexual sensation.

In a U.S. survey, a greater proportion of circumcised men than uncircumcised men reported being satisfied with their circumcision status [118]. Instead of accepting the findings at face value, the researchers suggested that neonatally circumcised men refuse to accept that they have been harmed by their NMC. Stephen Moreton, Ph.D., a British skeptic, found that not only was their interpretation of the findings fanciful, but the correct answer to some of the 10 “true/false” statements in the questionnaire was irrelevant, ambiguous, or incorrect, and that the authors had ignored the scientific evidence that NMC has no adverse effect on sexual function or erogenous sensation [127].

A survey by other NMC opponents of a cohort of men who already believed that their sexual function and experience had been diminished by NMC reached a conclusion that was consistent with the beliefs of that cohort [114]. A U.S. clinical psychologist, Stefan Bailis, Psy.D., and his colleagues in other fields, Moreton and Morris, suggested that the survey and its uncritical presentation may have been driven less by consideration of the scientific evidence and more by psychological factors, in that individuals who naïvely come to believe the anti-MC narrative that they have been harmed by their NMC may form a strong opinion about MC and would be more likely to participate in such a survey and respond to the questions in the way many did, thus introducing a risk of bias [122].

A survey of a general population sample of 744 U.S. Amazon Mechanical Turk survey participants, 408 of whom had been circumcised neonatally, was conducted by Alessandro Miani, M.A., Aarhus University in Denmark, and European and U.S. colleagues, one of whom was Brian Earp [201]. The survey investigated socio-sexuality and stress parameters using a battery of psychometric scales for 21 parameters. The conclusion was that NMC might have an impact on adult socio-affective traits or behaviors. A critical evaluation by Morris and colleagues found that after correcting the data for multiple testing by the Holm-Bonferroni method, only sociosexual desire (18% higher), dyadic sexual libido/drive (7% higher), and stress (14% higher) remained significant [202]. The critics speculated that the relatively greater sexual activity found in circumcised men might reflect reduced sexual activity in uncircumcised men overall owing to pain and psychological aversion in those with foreskin-related medical conditions (reverse causality). Since NMC was not associated with empathy in the participants, the critics pointed out that the data contradicted the hypothesis by NMC opponents that procedural pain during NMC causes central nervous system changes [202]. Miani et al. did, however, state that “the psychological differences that we found are not sufficiently severe in themselves to be suggestive of pathology.” The systematic review by Morris et al. also covered the entire spectrum of literature on the psychological effects of NMC, leading them to conclude that “the highest quality evidence suggests that neonatal and later circumcision has limited or no short-term or long-term adverse psychological effects” [202].

Views opposing NMC are abundant on the Internet and social media [136,203] and may dissuade some men with sexual problems from consulting a medical practitioner for advice and treatment [76,122]. Men with normal sexual function could also conclude that their sex lives have been diminished and, as a result, experience anxiety and resentment regarding their parents’ decision to have them circumcised. Indeed, given the finding of a strong correlation between depression and sexual dysfunction in a systematic review [96], the distress associated with the narrative of NMC opponents could adversely affect the sexual function and mental health of vulnerable men, though being well-informed about research findings should allay this effect [122].

Parents may decide to have a newborn son circumcised to ensure that his penis resembles those of his peers when he is older. A survey of undergraduate males conducted in the U.S. state of Iowa found that 87% were circumcised and that, of the teasing about their penises experienced in locker rooms, 75-83% concerned size and 24% concerned being uncircumcised [106]. A study conducted in Sweden, where MC is rare, found that among schoolboys circumcised for phimosis a decade earlier, slight shyness in the locker room was the only adverse psychological effect [84]. According to an extensive systematic review of all of the available evidence, the consensus in the highest-quality literature is that males circumcised either neonatally or in adulthood experience minimal or no adverse effect in terms of sexual function, sensation, or pleasure [196].

Biomedical misunderstandings

Opponents of the NMC Myers and Earp claimed that, in infancy, the foreskin is “fused” to the shaft skin and “degloving” (i.e., removal of shaft skin with the foreskin) occurs during NMC [59]. In reality, the foreskin can be gently separated from the underlying shaft skin early in the NMC procedure, so it would avoid degloving. The frequency of an open wound to the penis during medical NMC in the United States is very low: 0.0006% [100]. It is the case that small-scale studies in various countries, often involving ritual or traditional MC by individuals without medical training, have reported higher prevalences of adverse events, including severe complications [90].

Myers and Earp, who argued that “it is unethical for a doctor or other healthcare provider to handle a child’s genitals beyond what is strictly necessary for diagnosis or treatment,” suggested that the foreskin should not be removed at birth because the owner might later want to use it for the construction of a “neovagina” during gender reassignment surgery. However, only 12.5 per 100,000 such individuals seek a male-to-female procedure [204], a figure similar to the annual rate of deaths in motor vehicle accidents per 100,000 people in the U.S., and not all who do so want a neovagina, as other options are available for those who want one [205].

While NMC is generally performed on a healthy penis, there are other surgical procedures that would seem highly advisable for boys. An example is the correction of the not uncommon congenital defect of hypospadias, a condition that leads to difficulties with urination, physical problems, and later sexual problems [206]. Yet some individuals argue against surgery to repair hypospadias in boys [103]. Hypospadias repair is often unsuccessful, and this may be why NMC opponents use it as an analogy. Better analogies may be the treatment of micropenis with steroids and the treatment of undescended testis by transposition to the scrotum to prevent cancer and infertility. As with NMC, for all of these procedures, boys aged under five years have no recollection of the surgery [86].

There is also an argument against therapeutic MC for phimosis and penile inflammatory conditions in males of any age, despite its effectiveness as an immediate treatment for these [175,207]. Therapeutic MC also cures painful erectile difficulties and reduces the risk of other diseases over the lifespan, including penile cancer [190-192,208] and prostate cancer [209]. For the busy family physician, treatment of phimosis with topical steroid creams is simpler and, for the patient, cheaper. This option is supported by opponents because it means that the foreskin is preserved [59], although, while simpler than MC, the use of steroid creams is far from ideal, with success rates of 61%-68% by 12 weeks of treatment [207,210,211]. In particular, topical steroid treatment requires a commitment to regular application for an extended period and carries a risk of side effects when used long-term, as well as cataracts, to name just a few downsides [117]. For the very serious foreskin-related inflammatory condition of lichen sclerosis, which rises in uncircumcised boys to a prevalence of 0.4% by age 17 years [207, 212], a systematic review found steroid treatment for 1-23 months (median two months) was only 35% effective at 1.5-60 months follow-up and prevented the need for circumcision in only 35% of lichen sclerosis cases [210]. By contrast, treatment by MC was close to 100% effective [108]. Preputioplasty can be used to accommodate the wishes of those wishing to preserve the foreskin but is less effective as a cure than MC [124], which is usually suggested when, not uncommonly, lichen sclerosis recurs after preputioplasty [126].

It has been suggested that the risk of UTI in boys does not warrant preventative NMC because oral antibiotics can be used [59,62,64]. However, the oral administration of antibiotics to infants is difficult, and absorption is low, so hospitalization may be required for intravenous administration [102,213]. The emergence of resistance to most or all antibiotics, including methicillin, has increased the challenges associated with pediatric UTI treatment [214]. 

Hay [99] and Booker [49], in criticizing the cost-benefit analysis of NMC by Johns Hopkins researchers Seema Kacker and colleagues [170], argued that NMC for the purpose of preventing STIs is unethical because of the availability of vaccinations (against HPV) and prophylactic medications (against HIV). In response, Kacker et al. pointed out, among the misunderstandings in their arguments, that the uptake of vaccination and prophylactic medications has been low and that the cost-benefit analysis was conservative, “resulting in an underestimate of the true health and financial implications” of NMC for STI prevention [54]. Myers and Earp, however, argued that the risks from vaccination are trivial while those from MC are “catastrophic” since the procedure causes “genital skin laceration” [59]. In fact, as pointed out above, “laceration” is rare. The commonly used Plastibell device is based on compression rather than cutting, and the Gomco clamp is designed to ensure no hemorrhage occurs when the foreskin is excised.

Some opponents of MC, then, hold beliefs unjustified by scientific evidence and adhere to disproven or even fabricated [215,216] claims and speculative ideas [76]. As a general comment, the denial of scientific evidence is not new, but in the digital age, evidence-based conclusions are increasingly challenged by beliefs based on emotion and isolated anecdotes. Facts are disputed, with science deniers utilizing social media to air their views, presenting a challenge for scientists and society [116,135-140,153]. Opponents of NMC appear to be a subset of the diverse landscape of science deniers. As the late U.S. Senator Daniel Patrick Moynihan was fond of saying, “Everyone is entitled to their own opinion, but not their own set of facts” [217].

Psychological consequences of opposition to male circumcision

Faced with the barriers to MC at an older age (Table 2), uncircumcised males may wish that they had been circumcised in infancy. On the other hand, as discussed, circumcised males may conclude, based on some of the arguments against MC, that they are missing something by not having a foreskin. Foreskin restoration can be undertaken despite being described as “a very burdensome and time-consuming process that, if successful, creates a pseudo-prepuce only” [59]. The effort, if intended to increase sexual satisfaction, is futile, for, as discussed, most cohort studies comparing men who were circumcised with those who were not, irrespective of the age at which their MC took place, find their sexual satisfaction to be the same or greater [196]. It may be that the claims by opponents of NMC (e.g., [114,118]) could cause psychologically vulnerable circumcised men to question whether unrelated medical or psychological symptoms that they are experiencing are associated with their circumcision status and, consequently, to suffer undue anxiety while the cause of their symptoms remains untreated. Likewise, men who require circumcision for medical reasons may be deterred from consulting a medical practitioner, thus unnecessarily prolonging or exacerbating their condition.

Vaccination analogy

The similarities between opposition to NMC and vaccination include the fact that both involve a physical intervention on a neonate, infant, or child, pain (that can be reduced with the application of a local anesthetic), a low risk of adverse events, proxy consent given by the parents or guardians rather than the individual, and, in at least some cases, ignorance about the intervention and its consequences. Both also raise ethical and legal issues for some individuals. In the case of NMC, additional issues include the fact that minor surgery is involved and that the circumcision of older boys has the potential to fuel prejudice in most of Europe, Asia, and Latin America, where circumcision is largely confined to religious and cultural minorities. Further, the consequences of not circumcising or vaccinating include a greater likelihood of avoidable infections, suffering, and even death. Like circumcision, vaccination is an intervention, although rather than taking place once, many vaccines need to be administered multiple times, either over months or annually.

One study estimated that one UTI was prevented in infants for every 39 NMCs and for 29 NMCs when other sequelae were included [218]. By comparison, it has been estimated that one case of influenza is prevented for every 50 children who are vaccinated [219]. UTIs in infancy can result in significant morbidity (such as infection of the kidneys: pyelonephritis) and are the most common cause of sepsis in male neonates [195,220]. Within the first two years of life, the incidence of serious kidney conditions was five-fold higher in uncircumcised boys [218,221].

In defending the legality of NMC, Millard and Goldstuck [81] stated that “many of the preventive interventions that we routinely recommend for our patients have some degree of risk, but we recommend them when scientific evidence clearly demonstrates that the benefits outweigh the risks. Immunizations are a good example of this practice.” In the current era of vaccination against SARS-CoV-2, side effects have been well publicized but are generally accepted because the population benefits far outweigh the risks [222,223].

According to Hainz [46], “circumcision should be subsumed under human enhancement and treated like other enhancement technologies… [and that] prohibition appears to be unjustified because it would deprive parents of the possibility of providing their children with protection (although not total) against various diseases …. [and that] maybe circumcision is on a par with other interventions that alter the nature of humans but are usually regarded as permissible or even recommended, or obligatory, such as vaccination …. [furthermore, that] … The permanent and irreversible removal of the foreskin appears not to be less radical than a form of enhancement for children that is widely accepted: vaccination.” Hainz argued elsewhere that “ethically speaking, circumcision for religious intentions is unacceptable,” especially when some forms of religious circumcisions involve dangerous practices such as metztitzah b’peh (direct oral suction) [36]. Ben-Yami advocated minor changes to NMC, namely making anesthesia compulsory, having an upper age limit of a few months, and banning metztitzah b’peh [26].

Risk compensation

Table 3 lists STIs against which NMC protects. Condoms and other safe sexual behaviors provide additional protection. The suggestion that, once circumcised, men would forego condom use is contradicted by meta-analyses that found no difference in the use of condoms for up to two years after adult MC [129,224]. Unlike condoms, MC is a one-off procedure that does not require future compliance each time a man has sexual intercourse. Besides the hepatitis B vaccine, the only vaccines widely used for the prevention of an STI are those directed at up to 9 of the 20 HPV genotypes that infect the anogenital region. MC and condom use provide less than complete protection against STIs, but their effects are additive [225].

Public health advice generally favors the adoption of a package of proven preventive measures rather than a subset to maximize impact. HPV vaccination of girls and boys early in high school can reduce the risk of HPV infection and, in turn, cervical, penile, and oropharyngeal cancer, but the uptake is variable across settings [226]; current vaccines do not protect against all high-risk HPV genotypes, and the long-term durability of effectiveness remains to be assessed. Vaccination early in high school with the quadrivalent HPV vaccine directed at the two most common high-risk HPV genotypes (HPV-16 and HPV-18), that comprise approximately 70% of high-risk genotype prevalence [227], and the two most common low-risk genotypes could, under circumstances of 100% uptake (which is unrealistic) and efficacy, reduce high-risk genotypes by approximately 70%. Because these high-risk HPV genotypes are found in ~50% of penile cancers, HPV vaccination should, under the most optimistic of scenarios, lower the prevalence of penile cancer by 50 x 0.7 = 35% [93]. A systematic review found vaccination was associated with a 28% and 34% reduction in incident anogenital infections caused by HPV16 and 18, respectively [228]. Data from a meta-analysis showed an overall 32-33% level of effectiveness of MC in reducing high-risk HPV acquisition [229,230].

NMC policy statements

Morten Frisch and other NMC opponents from northern Europe criticized the 2012 AAP infant MC policy [5,6] for being “culturally biased” [98]. In response, articles by the AAP Task Force on infant male circumcision argued that the cultural bias was instead in Europe [68] and called for respectful dialogue [24]. The article by Frisch et al. implies that leaving circumcision up to parents is culturally biased, but not letting people whose culture demands circumcision obtain it is not culturally biased. It should be apparent that in any society, adults would have already adopted a body image and have been socialized by the majority society.

Darby singled out the AAP policy’s failure to factor in “the value of the foreskin to the individual and the physical and ethical harms of removing it from a non-consenting child” [73]. Morris, Krieger, and Klausner depended on “speculative claims about the foreskin and obfuscation of the strong scientific evidence” while failing to appreciate “the psychological, scheduling, and financial burdens later circumcision entails, so reducing the likelihood that it will occur” [143]. Svoboda and Van Howe claimed there were “fatal flaws” in the AAP’s 2012 policy statement [63]. Morris, Tobian, Hankins, Klausner, Banerjee, Bailis, Moses, and Wiswell disagreed, finding that the claim by those NMC opponents that the AAP’s policy was a "partisan excursion through the medical literature, improper analysis of available information, poorly documented and often inaccurate presentation of available findings, and conclusions that are not supported by the evidence” was actually applied instead to Svoboda and Van Howe’s critique, so that, if anything, the AAP’s policy “could be criticized for being too conservative" [58]. AAP Task Force member Michael Brady warned of the threat to “delivery of optimal healthcare to children … by those who have emotional or subjective beliefs about NMC” and against delay to “allow the male to participate in the decision-making process,” since “many of the health benefits that can be achieved by NMC are lost if the procedure is deferred to age 18 years” [30]. He pointed to the high frequency of sexual activity and acquisition of STIs by U.S. teens, with the CDC finding that 49% of twelfth-grade students had had sexual intercourse, including 18% having had more than four sexual partners [231]. He declared that “not providing parents with appropriate information concerning the health benefits of NMC would actually be unethical” and that “clearly parents consistently make decisions for their children based on what they believe is in the best interest of their child.”

Vogelstein asserted that, because the AAP’s stance was based largely on scientific data demonstrating that the benefits outweigh the risks, unlike its detractors, “the AAP plausibly has the bona fides to pronounce upon this issue, … [and is] … likely to be correct … [deeming the issue a] …. false controversy,” given the strength of the arguments in favor and weakness of the opposing arguments [67]. Van Howe [66] disputed Vogelstein’s stance and would have liked the AAP to address issues such as ethics and human rights, as well as the previous arguments Van Howe himself has published in opposing NMC [63], but did not mention that the latter arguments had already been repudiated by others [58].

The CDC’s review and recommendations on MC, comprising several documents issued together in 2018 [7,8], were accompanied by detailed responses to public comments on its 2014 draft statement [232]. Most of the responses were from opponents, who made unfounded claims such as failure to reference “the studies that have proven that there is no benefit to MC," that “the CDC is a culturally biased organization … making a culturally biased recommendation," that “there is insufficient evidence to make a recommendation for the U.S.” and naming several European countries as having “taken a stance against [NMC]”. The CDC responded extensively to all comments by referring to the evidence identified in their systematic review that contradicted the public comments and saying that “the literature review was updated through October 2015 during the process of responding to the peer review and public comments with corresponding updates made to the recommendations statement." Criticisms of the 2014 draft statement were published in non-PubMed or low impact factor journals by individuals such as Earp, Svoboda, Adler, and Van Howe using mostly legal, ethical, and human rights arguments [50,64,111,119]. Responses to these by medical, scientific, and bioethics experts Krieger, Klausner, Morris, Rivin, and Diekema published in the same journals pointed out the scientific, ethical, and legal flaws in each and how acceptance of the claims had the potential to undermine public health and individual well-being [75,109,113,133].

A policy on NMC by the Canadian Paediatric Society in 2016 [110] was found to be based on a risk-benefit analysis that did not include data for all common benefits and risks, nor did it calculate a benefit-to-risk ratio, meaning that its so-called risk-benefit analysis was flawed [112]. As a result, the position statement concluded only that “there may be a benefit for some boys in high-risk populations and circumstances where the procedure could be considered for disease reduction or treatment [and that] the Canadian Paediatric Society does not recommend the routine circumcision of every newborn male" [110]. The guidance of the British Medical Association (BMA) [233] was seen as being limited in scope, in that it focused mainly on religion, risks, and ethics, while claiming that, as a clinical body, it was not capable of reviewing the medical evidence [128]. Nevertheless, NMC opponents Lempert, Chegwidden, Steinfeld, and Earp criticized the BMA’s guidance [234]. Moreton et al. then pointed out the flaws in those criticisms, which included statements that NMC was high-risk, had little or no benefits, led to diminished sexual pleasure, was unethical, legally questionable, and should be delayed until the boy could decide for himself whether to get circumcised [235].

Policies on NMC emerged soon after the appointment of pediatricians from the U.K. to Chairs of Pediatrics in Australia and New Zealand, where they discovered that unlike in the United Kingdom, where NMC is principally performed on male children of the upper classes as a sign of having been delivered by a physician, children of lower-class families tend to be delivered by midwives, who do not perform circumcisions; instead, most boys bore the “mark” of the upper classes. It resulted in the newly appointed professors successfully changing the culture away from the almost universal practice of NMC in the 1950s and 1960s [131]. Over the years, successive NMC policies by the Royal Australasian College of Physicians (RACP) have discouraged the procedure, but with ever-strengthening evidence in favor, the 2010 RACP policy [236] was criticized by prominent Fellows of the RACP and other medical bodies who found the policy not to be evidence-based [95]. The Royal Dutch Medical Association’s policy, developed by an ethicist, describes NMC as a “violation of children’s rights,” refers only to the complications associated with the procedure, and urges “a strong policy of deterrence” [146]. Similarly, without any apparent consideration of the scientific evidence, the Danish Medical Association declared that non-therapeutic MC is “ethically unacceptable” [237].

The affirmative evidence-based policy recommendations of the AAP and the CDC emphasize disease prevention by safe NMC and MC at any age. The first evidence-based policy was that of the AAP in 2012, followed by the CDC’s draft policy in 2014 and the CDC’s final recommendations in 2018. These policies have now raised the bar, meaning that medical organizations globally should no longer rely on opinions but must now consider the extensive, high-quality scientific evidence as an integral part of developing policies and recommendations relating to NMC. The trend in NMC policy statements appears to have followed the trend that took place in medical teaching early this century towards evidence-based evaluation for clinical decision-making. It should, therefore, come as no surprise that, because of the accumulating evidence, NMC policies in the past decade have become increasingly supportive of NMC for families who choose it.

Limitations

This systematic review did not use controlled vocabulary terms such as MeSH (Medical Subject Headings) and Emtree (a collection of standardized keywords in Embase) in the search strategy because of the enormous number of irrelevant publications that would result from the single keywords. Instead, we used “circumcision male” combined with “ethical", “ethics,” or “legal” for PubMed searches and similar terms as appropriate for the methods adopted by the other databases searched. Many of the references retrieved could be categorized as arguments reflecting societal views and opinions and the writers’ cultures, peer groups, standing (academic or lay), and/or allegiance to groups that oppose or support NMC.

## Conclusions

This systematic review finds: (1) arguments against NMC are mostly based on low-quality evidence in support of it being high-risk, has little or no health benefit, causes psychological harm, impairs sexual function and pleasure, is unethical, and may be illegal; (2) arguments in favor of NMC are based on evidence that is generally high-quality, showing substantial wide-ranging health benefits, is low-risk, does not cause psychological harm, has no adverse effect on sexual function or pleasure, and is ethical and legal. On balance, the findings presented indicate that parent approved medical NMC and indeed MC at any age, by competent practitioners is ethical and consistent with the right to health. A decline in NMC rates, irrespective of the cause, would increase adverse medical conditions and the burden on healthcare systems. NMC is recognized by major, well-informed health authorities as an important public health intervention that is both ethical and legal. These findings thus have significant implications for clinical practice, public health policies, and future research.
